# Fatty Acid Profile Is Modulated by Dietary Resveratrol in Rainbow Trout (*Oncorhynchus mykiss*)

**DOI:** 10.3390/md15080252

**Published:** 2017-08-11

**Authors:** Claudia Torno, Stefanie Staats, Sonia de Pascual-Teresa, Gerald Rimbach, Carsten Schulz

**Affiliations:** 1 GMA-Gesellschaft für Marine Aquakultur mbH, Hafentörn 3, 25761 Büsum, Germany; 2 Institute of Animal Breeding and Husbandry, University of Kiel, Olshausenstraße 40, 24098 Kiel, Germany; cschulz@tierzucht.uni-kiel.de; 3 Institute of Human Nutrition and Food Science, University of Kiel, Hermann-Rodewald-Straße 6, 24118 Kiel, Germany; staats@foodsci.uni-kiel.de (S.S.); rimbach@foodsci.uni-kiel.de (G.R.); 4 Department of Metabolism and Nutrition, Institute of Food Science, Food Technology and Nutrition (ICTAN-CSIC), José Antonio Novais 10, 28040 Madrid, Spain; soniapt@ictan.csic.es

**Keywords:** CPT1, fish oil replacement, hepatic fatty acid synthesis, long chain polyunsaturated fatty acids, mRNA expression, phytochemical, PPARα

## Abstract

To produce fish of a high quality that are rich in omega-3 fatty acids (n-3 FA) and simultaneously generate more sustainable aquaculture, the combined use of phytochemicals and vegetable oils in fish feed seems to be a promising approach. Resveratrol (RV) potentially induces endogenous fatty acid synthesis, resulting in elevated n-3 FA levels in fish. RV putatively influences ∆6-desaturase, the key enzyme in FA metabolism, and serves as a ligand for PPARα, a transcription factor regulating β-oxidation. Rainbow trout (36.35 ± 0.03 g) were randomly allocated into six groups and fed diets with reduced fish oil levels (F4 = 4%, F2 = 2% and F0 = 0% of dry matter) supplemented with 0.3% (*w*/*w*) RV (F4 + RV, F2 + RV and F0 + RV). RV significantly affected FA composition in liver tissue and whole fish homogenates. 20:5n-3 (EPA) and 22:6n-3 (DHA) were significantly increased whereas precursor FA were diminished in fish fed the F2 + RV and F0 + RV diets when compared to F4 + RV and F0. RV significantly elevated ∆6-desaturase protein levels in the livers of F0 + RV fed animals. Hepatic mRNA expression of ∆6-desaturase, PPARα, and its target genes were affected by the dietary fish oil level and not by dietary RV. The results of this study indicated a potential benefit of supplementing RV in fish oil deprived diets elevating n-3 FA levels in rainbow trout.

## 1. Introduction

Seafood, fish, and fish products are important dietary sources of health beneficial omega-3 fatty acids (n-3 FA) and thus important in human nutrition [1,2,3,4]. In particular the n-3 long chain poly-unsaturated FA (LC-PUFA) eicosapentaenoic acid (EPA, 20:5n-3) and docosahexaenoic acid (DHA, 22:6n-3) may mediate health benefits and are of increasingly recognized importance [5,6,7,8]. For the production of healthy fish rich in these n-3 LC-PUFAs, the nutrition of the fish itself plays a crucial role [9]. On the one hand, marine ingredients in fish diets have a positive effect on fish health, survival and growth rates [10,11]. On the other hand, the shortage of marine resources for fish meal and fish oil production and the demand for sustainable aquaculture requires the use of alternative raw materials. These are often plant based and differ in nutritional value, digestibility and nutrient availability in comparison to marine derived feed ingredients. When dietary fish oil is replaced by plant oils, the resulting FA composition in fish often changes to a profile rather poor in n-3 FA, thus making them less valuable for human nutrition [12,13,14,15,16,17]. Considerable effort has been made in the past decade to counteract this trend where various approaches have been considered including the use of n-3 FA rich finishing diets after feeding plant based diets [14,18], the inclusion of microalgae rich in n-3 FA [19,20,21], the use of n-3 enriched oil from genetically modified oilseeds [22], and the application of secondary plant compounds to alter endogenous n-3 FA synthesis [23,24], amongst others.

**Figure 1:**
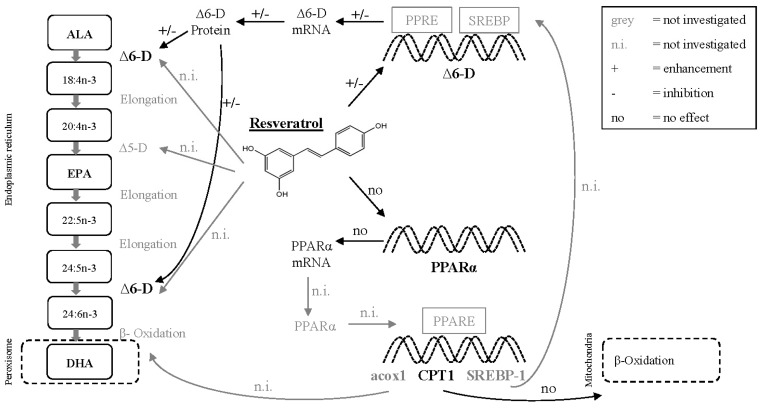
Molecular pathways of the hepatic endogenous fatty acid synthesis in the endoplasmic reticulum and peroxisomes and putative effects of resveratrol (RV) on the expression level and activity of involved enzymes and transcription factors. Alpha-linolenic acid (ALA) is converted to eicosapentaenoic acid (EPA) and further to 24:6n-3 via elongation and desaturation (∆6-desaturase (∆6-D) and ∆5-desaturase (∆5-D)) steps [26]. The partial β-oxidation to form docosahexaenoic acid (DHA) takes place in peroxisomes. RV possibly affects ∆6-D and ∆5-D activity, or interacts with ∆6-D via transcriptional control of gene expression [48]. Further, RV putatively enhances the peroxisome proliferator-activated receptor α (PPARα) gene expression [46,47]. PPARα might induce the expression of target genes, for example carnitine palmitoyl transferase 1 (CPT1), sterol regulatory element binding protein 1 (SREBP-1) and acyl-CoA oxidase 1 (acox1) [37,38], putatively increasing mitochondrial β-oxidation (CPT1), peroxisomal β-oxidation (acox1) and ∆6-D gene expression (SREBP-1). Graph made according to Sargent et al. [26] and Burdge [28] and modified according to results from previous studies [37,38,46,47,48] and own data.

Fresh water fish have the ability to synthetize EPA and DHA [25,26,27] from the essential precursor n-3 PUFA alpha-linolenic acid (ALA, 18:3n-3). The hepatic FA conversion from ALA to EPA and further to DHA takes place in the endoplasmic reticulum and comprises several elongation and desaturation steps, followed by a final β-oxidation in the peroxisome [26,28] (Figure 1). The LC-PUFA synthesis is a complex process requiring different enzymes, transcription factors and various target genes. The rate-limiting key enzyme of this process is ∆6-desaturase (∆6-D), which introduces an additional double bond in ALA and initiates the LC-PUFA synthesis [29]. Aside from the ∆6-D and ∆5-desaturase (∆5-D), the nuclear hormone receptors peroxisome proliferator-activated receptors (PPARs) and its target genes including carnitine palmitoyl transferase 1 (CPT1), sterol regulatory element binding protein 1 (SREBP-1), and acyl-CoA oxidase 1 (acox1) are centrally involved in LC-PUFA synthesis and regulation of this process (Figure 1). 

In rainbow trout (*Oncorhynchus mykiss*), the endogenous FA synthesis may last 2–4 weeks to convert dietary ALA into EPA and DHA and contributes only to a small extend to n-3 LC-PUFA tissue levels [30]. This indicates that LC-PUFAs are putatively essential for rainbow trout and should be supplied via dietary intake. Under experimental conditions, it is feasible to reduce marine feed ingredients (and thus dietary EPA and DHA levels) without compromising fish performance [31,32]. However, a more recent study showed that a complete reduction of marine feed ingredients over a whole production cycle in rainbow trout was possible [33]. However, the vegetable diet significantly negatively affected growth and the n-3 FA content of fish (carcass n-3 PUFAs in polar lipids reduced from 45.1% to 32.1% FAMEs and in neutral lipids from 31.4% to 14.3% FAMEs) [33]. Thus, in commercial scale production, trout diets still need to meet minimum requirements for essential fatty acids (EPA + DHA: 0.4–0.5% DM of diet and ALA: 0.7–1.0% DM of diet [34]) to ensure an end product with adequate amounts of n-3 LC-PUFA. According to several studies, the n-3 LC-PUFA content of farmed and wild salmonids varies largely depending on commercial feed and/or region (rainbow trout farmed versus wild: 931 and 268 mg/100 g, respectively [35]; Atlantic salmon farmed vs. wild: >1400 and <900 mg/100 g, respectively [17]). In farmed salmonids, amounts of EPA and DHA are decreasing parallel to increasing use of dietary vegetable oils leading to increased portion sizes to meet recommended dietary amounts of EPA + DHA of 200–500 mg/day [17,36]. For the production of healthy fish with sufficient amounts of EPA and DHA and a reduction of marine dietary resources, secondary plant compounds that may have the ability to modify endogenous FA synthesis in vivo should be considered. Genistein and lipoic acid [37], or a mixture of episesamin/sesamin [37,38] were shown to increase the expression levels of genes encoding proteins involved in FA synthesis in salmon hepatocytes in vitro (for example ∆6-D, PPARα and CPT1). Further, Trattner et al. [23] enhanced EPA and DHA levels in rainbow trout in vivo with feeding diets containing a sesamin/episesamin mixture. EPA was elevated significantly from 5.2% to 5.6% FAMEs and DHA from 38.6% to 43.7% FAMEs when rainbow trout were fed sesamin/episesamin-supplemented diets [23].

In this study, the phytochemical resveratrol (RV) was investigated for its potential to induce LC-PUFA synthesis in rainbow trout. RV is a stilbene found in different plants, mainly grapevine, with potentially health-beneficial and, amongst others, anti-oxidant and anti-inflammatory properties [39,40,41]. Over thirty years ago, RV was proven to modulate lipid metabolism (reduced lipogenesis and increased lipolysis) in rats [42,43]. RV up-regulated PPARα in a neuron model [44] and furthermore was described as PPAR activator in several in vitro and in vivo studies, as reviewed by Nakata et al. [45]. Two studies, both combining in vitro cell culture assays with in vivo experiments in mice, suggested that RV activated PPARα [46,47]. RV putatively increased elongase and desaturase activities (∆6-D and ∆5-D) resulting in elevated DHA levels [48] (Figure 1). Recently, polyphenol-rich wine lees extracts were shown to modulate FA metabolism and FA profile in zebra fish embryos [49]. To the best of our knowledge, this is the first study investigating the potential effects of RV on endogenous FA synthesis in rainbow trout in vivo.

To investigate how the phytochemical RV may affect n-3 LC-PUFA synthesis, feeding experiments with rainbow trout were conducted. Experimental diets with RV supplementation and differing fish oil inclusion levels and thus, varying dietary LC-PUFA contents were used. The resulting FA composition of liver tissue and whole body homogenates, the hepatic mRNA steady state levels of ∆6-D, PPARα, CPT1a and CPT1c and hepatic protein levels of ∆6-D were determined. 

## 2. Results

### 2.1. Resveratrol Impairs Body Weight Gain in Rainbow Trout

At the end of the feeding trial, the mean body weight (FBW) of trout fed the un-supplemented basal diets (F4, F2, F0) was significantly higher compared to animals fed diets supplemented with RV (F4 + RV, F2 + RV and F0 + RV; Table 1, *p* < 0.05 indicated by letters). The daily feed intake (DFI) was significantly (*p* < 0.05) reduced in trout fed the RV-supplemented diets compared to un-supplemented control diets. The hepatosomatic index (HSI) varied between 1.28 ± 0.30% (F2 + RV) and 1.52 ± 0.40% (F4) and did not significantly differ among groups. 

### 2.2. Experimental Diets Affect the Fatty Acid Composition in Whole Body Homogenates of Rainbow Trout

The FA composition of the diets (Table 2) was equally altered in the basal diets as in the corresponding RV supplemented diets with decreasing fish oil content. In general, monounsaturated FA (MUFA) content increased with decreasing fish oil content, while saturated FA (SFA) and PUFA contents decreased. Predominantly, the absolute amounts of EPA + DHA % DM of diet decreased with decreasing fish oil content from approximately 0.8% DM (F4 diets) to approximately 0.3% DM (F0 diets). At the same time, the ratio of ALA (18:3n-3) to LA (18:2n-6) remained unaffected (0.32–0.38) in all diets (Table 2).

The FA composition of the whole body homogenates of rainbow trout was not modified following a reduction of fish oil content while increasing the vegetable oil content in the basal diets (F4, F2 and F0; Table 3). Nevertheless, significant increases of EPA and DHA concentrations and simultaneous significant decreases of 18:2n-6 (LA) and 18:3n-3 (ALA) levels were only detected in whole fish homogenates when trout were fed the RV-supplemented diets (+RV; Table 3, *p* < 0.05 indicated by capital letters A, B). In RV-treated animals, tissue EPA + DHA levels significantly (*p* < 0.05) increased from 21.29 ± 7.29% (F4 + RV) to 32.63 ± 0.89% (F2 + RV) and 38.35 ± 3.38% (F0 + RV). The total amount of PUFA was also significantly (*p* < 0.05) increased from 38.41 ± 3.91% (F4 + RV) to 47.60 ± 1.55% (F0 + RV). The n-3/n-6 ratio was significantly (*p* < 0.05) increased from 2.34 ± 1.45 in fish fed the diet F4 + RV to 8.24 ± 2.61 in fish fed the F0 + RV diet.

When the FA profiles of rainbow trout that were provided with equal dietary fish oil levels, but either +RV or no supplementation with RV were compared, significant differences were most apparent in fish fed the fish oil free diets (F0 and F0 + RV). Concentrations of most FA changed in response to dietary supplementation with RV when compared to the controls (Table 3, *p* < 0.05 indicated by letters a, b and x, y). Most noticeably, DHA levels increased from 19.24 ± 2.33% in the controls (basal diet F0) to 32.81 ± 3.11% in the RV-treated animals (F0 + RV). Dietary supplementation with RV led to a significant (*p* < 0.05) increase in the contents of EPA + DHA, as well as total PUFA in the whole body homogenates of fish fed F2 + RV and F0 + RV diets. The n-3/n-6 ratio significantly (*p* < 0.05) increased from 2.42 ± 0.57 in fish fed the F0 diet to 8.24 ± 2.61 in fish fed the F0 + RV diet. No effect on FA profile was observed in groups which received the reference diets (F4 and F4 + RV).

### 2.3. Experimental Diets Affect Lipid Levels and Fatty Acid Composition in Livers of Rainbow Trout

The liver lipid levels (total fat mg/g) of rainbow trout fed the six experimental diets were altered by the dietary fish oil inclusion level and the dietary supplementation with RV (Table 4). The reduction of dietary fish oil from 4% DM to 0% DM led to a significant increase in the liver lipid levels (Table 4, *p* < 0.05 indicated by letters M, N). Furthermore, in the RV-treated animals, the liver lipid levels were significantly increased compared to the control animals (F4 + RV in comparison to F4 and F2 + RV in comparison to F2, Table 4, *p* < 0.05 indicated by letters m, n and x, y, respectively).

The dietary treatment only slightly affected the relative FA profile (% FAMEs) in the livers of rainbow trout. The amount of SFA was decreased in fish fed the F0 + RV diet compared to the basal F0 diet (*p* < 0.05 indicated by letters a, b), whereas the MUFA and the PUFA contents did not change based on dietary treatment. When the liver lipid levels are taken into account, the absolute amounts of EPA + DHA were significantly increased in fish that were fed the F0 diet compared to F4 (Table 4, *p* < 0.05 indicated by letters M, N). In the RV-treated fish, the absolute amounts of EPA, DHA and EPA + DHA were significantly (*p* < 0.05) elevated when fish were fed the F2 + RV and the F0 + RV diets compared to the un-supplemented basal diets. The absolute amounts of EPA + DHA were elevated from 8.69 ± 1.78 mg/g (F0) to 12.38 ± 2.93 mg/g (F0 + RV, Table 4, *p* < 0.05 indicated by letters a, b) in the liver tissues. No effect on FA profile was observed in groups which received the reference diets (F4 and F4 + RV).

### 2.4. Dietary Fish Oil Content Affects Hepatic mRNA Expression Levels

**Figure 2:**
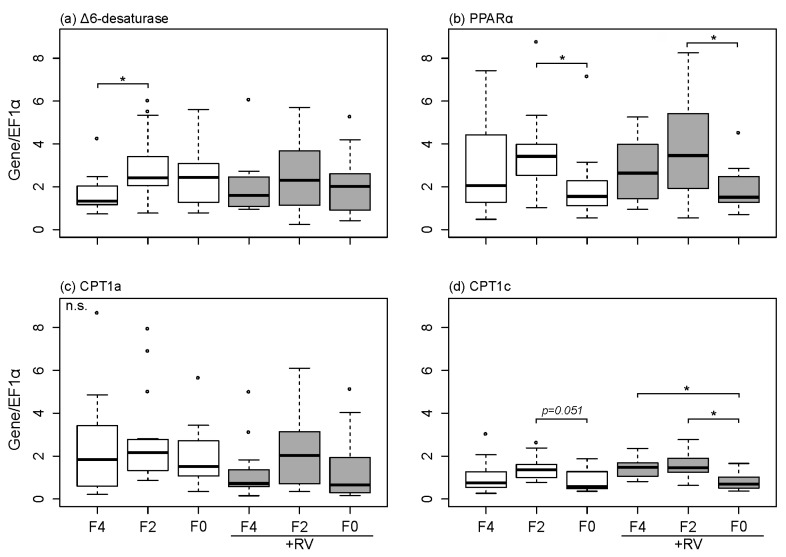
Hepatic mRNA expression levels in rainbow trout liver following dietary treatment with varying levels of fish oil and resveratrol supplementation for eight weeks. (**a**) ∆6-desaturase; (**b**) PPARα; (**c**) CPT1a and (**d**) CPT1c were measured in the liver of fish using qRT-PCR and were normalized to the housekeeping gene EF1α. Feeding groups F4, F2 and F0 were fed basal diets containing 4%, 2% and 0% DM fish oil, groups with +RV were fed respective diets supplemented with resveratrol. Boxes represent values (*n* = 15) between the 25 and 75 percentiles; whiskers indicate 1.5 SD; the solid line indicates the median; circles represent values above and below SD. Significant differences (*p* < 0.05) were analyzed using multiple contrast tests for relative effects. Tests were based on comparisons of fish oil inclusion level within one supplement group (indicated by *) or supplement type within one fish oil inclusion level (no significant differences).

The effect of dietary treatments (fish oil level and supplementation of plant bioactive compound) on mRNA expression levels of ∆6-D, PPARα, CPT1a and CPT1c were investigated in the rainbow trout livers (Figure 2). 

The reduction of fish oil content in the diet significantly increased the hepatic mRNA expression levels of ∆6-D in fish following the eight week feeding period (F4 compared to F2, Figure 2a, *p* < 0.05 indicated by *). Furthermore, hepatic PPARα mRNA expression levels were modulated by the different dietary fish oil levels (Figure 2b). Fish fed the F0 and F0 + RV diets displayed significantly (*p* < 0.05) lower PPARα mRNA expression levels when compared to fish fed the F2 and F2 + RV diets. This effect was independent of RV supplementation. Similar results were observed for the PPARα target gene expression CPT1c (Figure 2d). Hepatic mRNA expression of CPT1c was down-regulated when fish were fed the F0 diet in comparison to F2. When fish were fed diets supplemented with RV, the mRNA expression levels of CPT1c were significantly down-regulated in the group fed the F0 + RV diet when compared to F2 + RV and F4 + RV (Figure 2d, *p* < 0.05 indicated by *). Expression of CPT1a was neither influenced by dietary fish oil level nor dietary RV.

### 2.5. Resveratrol Affects Hepatic ∆6-Desaturase Protein Levels

**Figure 3:**
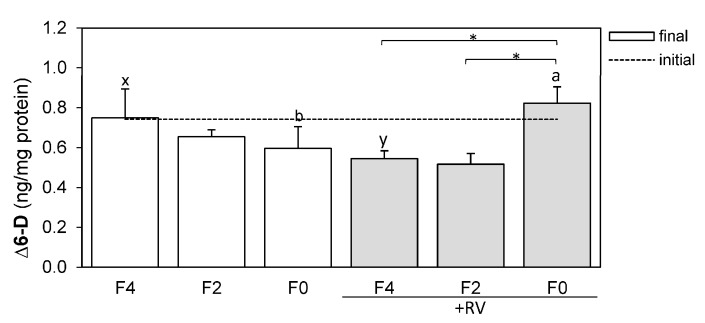
∆6-desaturase (∆6-D) protein levels in the livers of rainbow trout following dietary treatment with varying levels of fish oil and resveratrol supplementation for eight weeks. The ∆6-D levels were measured using ELISA and were normalized to the total protein level (ng/mg protein). Feeding groups F4, F2 and F0 were fed basal diets containing 4%, 2% and 0% DM fish oil and groups +RV were fed respective diets supplemented with resveratrol. The dashed line indicates the initial expression value (*n* = 1), bars indicate the final expression values (mean + SD, *n* = 3). Statistically significant differences (*p* < 0.05) were analyzed using multiple contrast tests based on comparisons of fish oil inclusion level within one supplement group (*) or supplement type within one fish oil inclusion group (a, b: F0 diets; x, y: F4 diets).

Protein levels of ∆6-D were determined in the livers of rainbow trout fed different dietary fish oil levels and in the presence or absence of the dietary plant bioactive compound RV (Figure 3). Feeding the RV supplemented diets significantly affected hepatic ∆6-D protein levels in trout. ∆6-D protein levels were significantly increased in the livers of fish fed a diet completely lacking in fish oil (F0 + RV) when compared to fish fed the F4 + RV and F2 + RV diets (Figure 3, *p* < 0.05 indicated by *). The ∆6-D protein levels of fish fed the F0 + RV diet (0.823 ± 0.082 ng/mg protein) were equally high when compared to fish fed the control diet F4 (0.749 ± 0.145 ng/mg protein). Additionally, ∆6-D protein levels were significantly increased in the livers of fish fed the F0 + RV diet when compared to their F0 fed counterparts (Figure 3, *p* < 0.05 indicated by a, b). When dietary fish oil is 4%, RV even decreases the ∆6-D protein levels (F4 and F4 + RV, Figure 3, *p* < 0.05 indicated by x, y).

## 3. Discussion

The discrepancy between (1) the need for constant high quality products with (2) the concurrent increasing demand for fish meal and fish oil for aquaculture production of fish for human nutrition accompanied by (3) the shortage of marine resources for fish oil production is a well-known yet unsolved problem. This study focused on the impact of varying fish oil concentrations in fish diets (4%, 2% and 0% DM) in the absence or presence of the plant bioactive resveratrol (RV, 0.3% of DM) on n-3 long-chain PUFA synthesis in rainbow trout. The current study revealed positive effects of dietary RV on the PUFA content in the livers and the whole body homogenate of rainbow trout, including underlying molecular mechanisms. 

The substitution of fish oil by vegetable oils and the provision of alternative, plant-derived protein sources in the fish diet is a common practice in salmonid and trout aquaculture. This “policy of replacement” has often caused alterations in the fatty acid composition in fish tissues [13,15,16,27,50,51] as dietary FA composition is strongly correlated to the fish tissue FA profile [11,12]. Observations made during this study only partly agree with previous studies. SFA, MUFA and PUFA contents, as well as single FA concentrations varied within basal diets, but stayed consistent in the whole body homogenates of fish independent of diet (Table 3). Emery et al. [52] showed that FA tissue composition did not necessarily reflect dietary FA composition, which may be related to an active in vivo FA metabolism. Furthermore, Sissener et al. [53] suggested that it may be too simple to presume that the body FA profile reflects the dietary FA composition since different FA are diversely incorporated due to their suitability for functional capacities and energy supply. Moreover, the diet formulation used in this study was intentionally reduced in dietary EPA and DHA content (EPA + DHA levels in diets: 0.26–0.91% of DM of diet). In the above-mentioned studies, diets were always formulated to meet the requirements of EPA and DHA in trout [34]. Comparison of the final and initial PUFA contents in fish tissue disclosed a reduction in LA, ALA and EPA concentrations (diets F4, F2 and F0). Simultaneously, an increase in DPA (22:5n-3) and DHA concentration was prevalent. This led to the assumption that the generally predominant vegetable based formulation of all diets evenly affected the n-3 FA synthesis regardless of the dietary fish oil level. Furthermore, trout fed a more vegetable based diet (F2) had significantly increased mRNA expression levels of ∆6-D compared to F4 fed animals. This was in accordance with previous studies on rainbow trout and salmonids [11,24,29]. Nutritional modulation of activity and expression of ∆6-D has also been widely observed in teleost fish [29] and in rainbow trout the maintenance of specific DHA levels was realized by the adaptation of desaturase activities [11], especially when the diet lacked these LC-PUFA. 

The dietary administration of resveratrol (+RV) resulted in a reduction of the final body weight (FBW), which was predominantly caused by a lower feed intake (DFI) in these fish (Table 1). Since fish were fed to apparent satiation, the reduced DFI was an indication for lower appetite and earlier satiety. This effect was only visible in RV-treated groups and not the groups with reduced dietary fish oil (F2 and F0). Therefore, this indicates the lower palatability of RV-supplemented diets and no effect of dietary n-3 LC-PUFA levels in general. The lower palatability of fish diets may be caused by feed additives and secondary plant compounds as reviewed by Ajiboye et al. [54]. Growth depression and reduced feed intake as a result of dietary administration of secondary plant compounds has been described for Chinook salmon and rainbow trout [55]. 

Nevertheless, the RV-supplementation of the experimental diets F2 and F0 (2% and 0% DM fish oil) significantly elevated EPA, DPA and DHA contents in trout tissue when compared to feeding with the respective basal diets. Simultaneously, ALA and LA levels were decreased in fish fed the +RV supplemented diets. An increase in EPA in the triglyceride fraction of zebra fish embryos in response to RV-rich wine lees had been reported by Caro et al. [49] and partly supports the findings in this study. In a study with rainbow trout of similar size (IBW of 34 g) that were fed with diets lacking in fish oil, the dietary administration of the phytochemicals episesamin and sesamin increased relative tissue EPA and DHA contents (% FAMEs) to levels that are comparable to this study [38]. In both studies, the EPA amount was increased to approximately 5.5% FAMEs, and the DHA amount was increased to approximately 43.8% FAMEs in the study by Trattner et al. [38] and to 32.8% FAMEs in this study. These results indicate a similar potential of RV in rainbow trout fed diets reduced in fish oil when set into the context described by the other study. 

From a scientific point of view, the FA composition of the liver is of great interest, because endogenous FA synthesis mainly takes place in the liver [56]. The dietary treatments (fish oil reduction and supplementation with RV) affected the liver lipid levels and the absolute amounts of EPA and DHA, whereas relative amounts of the FA remained un-changed. A dietary induced change in liver lipid levels is common in salmonids [11,23,38,57,58]. More interestingly, in this study, the increase in the liver lipid levels led to the increase of absolute amounts of EPA and DHA, which are increased further when dietary RV is given (Table 4). RV led to the elevation of DHA in rat hepatocytes [48], and polyphenol-rich wine lees elevated DHA in zebra fish embryos [49]. Studies with different model species or cell cultures prove the ability of polyphenols to interact with the hepatic lipid metabolism and FA synthesis. For example, an episesamin/sesamin mixture led to elevated DHA amounts in salmon hepatocytes [38], and provinol modified the quantity of liver FA in rats [59]. In general, the FA compositions of the liver and the whole body homogenate of rainbow trout did not represent the dietary FA composition, but showed clear influence of RV.

Furthermore, the RV-mediated elevation of DHA in the rat hepatocytes, was ascribed to increased elongase and desaturase activities of ∆5-D and ∆6-D [48]. In accordance with these findings, the ∆6-D protein level significantly increased in the livers of fish fed the F0 + RV diet compared to F0, F4 + RV and F2 + RV fed animals (Figure 3). Overall, this indicates that a RV-dependent enhancement of desaturation activity when dietary fish oil content is low, is apparent in the increased abundance of the ∆6-D enzyme. The hepatic ∆6-D protein levels were elevated in vivo by dietary RV in the F0 + RV fed fish to a level equal to the untreated controls (diet F4, Figure 3). The RV-mediated increase of the ∆6-D protein level at 0% dietary fish oil, but decrease of the ∆6-D protein level at 4% dietary fish oil can be explained by a negative feedback. When dietary EPA and DHA is sufficient (in this study 0.74–0.91% DM), the further synthesis of EPA and DHA seems to be stopped by RV. Only when the dietary EPA and DHA levels are limited (in this study 0.26–0.56% DM), the FA synthesis is enhanced by RV. This indicates the effect of RV on the key enzyme of FA bioconversion that finds expression in elevation of the enzyme itself, as well as EPA and DHA levels in the livers and the whole body homogenate of trout, when dietary n-3 LC-PUFA are insufficient to meet the requirements, and can be regarded as a qualitatively important output. 

In contrast, the ∆6-D mRNA expression levels were not increased by RV. The phenomenon of a lack of correlation between ∆6-D mRNA and protein levels was also described by Schiller Vestergren et al. [24] and Emery et al. [52]. Generally, mRNA expression is very sensitive and rapidly responds to dietary modifications [37]. Certain phytochemicals, e.g., sesamin and genistein exhibited a time-dependent activation of PPARα and CPT1 mRNA expression in vitro [37,38]. Thus, in this study, the enhancement of ∆6-D transcription was possibly not detectable in the liver samples taken on the second day after the last feeding. Furthermore, post-transcriptional modifications could also lead to increased mRNA stability [60], resulting in elevated protein levels independent of changes to mRNA levels. The bioavailability of the phytochemical RV was considered to be rather low [61] as RV is quickly metabolized, conjugated (by glucuronidation and sulfation [62]), excreted and thus not necessarily available in the tissues over a longer period of time [63]. Our results suggest that though there might be low availability of RV, the phytochemical is bioactive and exhibits effects on the fatty acid profile even at low concentrations. Additionally, RV might not only elevate LC-PUFA levels by modifying long chain PUFA conversion on a molecular level, but also through the protection of polyunsaturated fatty acids from oxidation via cell signaling. There is a consistent body of evidence for the antioxidant properties of RV [64,65], especially highlighted in a study focusing on the effect of wine polyphenols in human nutrition [66]. Antioxidant activity could be mediated by the induction of endogenous antioxidant defense mechanisms that are partly under the control of the transcription factor Nrf2 in humans and mice [67]. Wang et al. [68] also recently showed that RV induced the Nrf2 target gene glutathione peroxidase 4 in laboratory rodents, thereby protecting against oxidative stress.

Dietary administration of RV did not significantly modify hepatic PPARα mRNA expression levels (Figure 2b). PPARα is a transcription factor which is involved in the regulation of the expression of numerous target genes involved in FA bioconversion and was modified by RV in vitro and in vivo in mice [46,47]. Thus, aside from PPARα mRNA quantification, the mRNA expression levels of selected target genes should also be considered. Administration of RV did not change the hepatic CPT1a and CPT1c mRNA expression. Both CPT1a and CPT1c are located in the mitochondria and involved in regulating the rate of PUFA β-oxidation [38,69]. The results presented in this study indicated a general regulation of mRNA expression of PPARα, CPT1a and CPT1c by dietary fish oil level regardless of dietary RV. These findings are supported by a study in zebra fish embryos where polyphenol and RV rich wine lees failed to modulate PPAR expression [49]. Furthermore, findings by Vauzour et al. [70] indicated that anthocyanidins may not affect FA composition or modify molecular mechanisms of n-3 FA bioconversion. The mechanism of how RV possibly interacts with transcription factors or genes exhibiting a PPAR binding site is yet to be fully understood [71]. Furthermore, RV presumably interacts via mechanisms not investigated in this study. It is well known that RV not only interacts with the FA synthesis, but the hepatic lipid metabolism in general [48,72,73]. Especially the sirtuin mediated pathway in the livers of different model species is greatly affected by RV (for example, mice and humans [73], and zebra fish [74]).

Whether RV activates or inhibits distinct signal transduction pathways seems to mainly depend on the dose which is applied in vitro and in vivo. Many phytochemicals and dietary supplements exhibit dose-dependent effects for example, *trans*-ε-viniferin, *cis*-viniferin and gnetin H show dose-dependent cytotoxicity against mouse cancer cell lines in vitro [75]. Additionally, doses of 10 µM genistein, daidzein, and glycitein inhibited estrogen metabolism in primary liver cell culture from Atlantic salmon, lake trout and rainbow trout, whereas lower concentrations had no effect compared to the controls [76]. Dietary vitamin E supplementation also showed a dose-dependent mode of action and prevented tissues from lipid oxidation when administered at doses above 100 mg/kg to red sea bream [77] and salmon [78]. In this study, RV was administered at doses of 3 g/kg, which represented the upper margin of previously applied concentrations in fish [79,80,81,82]. The described effects may help to understand the lack of effects on the expression of certain genes (PPARα, CPT1a and CPT1c), but increased protein abundance of ∆6-D and elevated PUFA contents. Hypothetically, the administered dose of 3 g/kg (RV) might possibly exhibit only minor effects on the level of gene expression, but over the duration of the eight week trial led to increased amounts of the key enzyme in the LC-PUFA synthesis on the protein level. At the same time, protection of LC-PUFAs from oxidation possibly via the induction of endogenous antioxidant enzymes elevated their relative amounts.

The main aim of this study was a mechanistic approach to investigate how the phytochemical RV may affect n-3 LC-PUFA synthesis. In rainbow trout, it seems that RV generally shows a great impact on FA synthesis, regardless of the actual bioavailability. It seems to be the case that once RV reaches the liver of rainbow trout, it interacts with the key enzyme of FA synthesis, ∆6-D. Whether this interaction is directly or via transcriptional control, cannot be fully explained with the results obtained in this study. No effect of RV on the mRNA expression of ∆6-D, PPARα, CPT1a, and CPT1c could be detected with the methods applied. Nevertheless, the elevated amounts of ∆6-D protein in the livers of fish fed diets with insufficient amounts of EPA and DHA and supplemented with RV are in accordance with increased absolute amounts of hepatic EPA and DHA. Furthermore, also the FA profile of the whole body homogenate of these fish shows elevated amounts of EPA and DHA. Based on literature data and own observations, the processes behind RV mediated activation of the FA synthesis are most likely via transcriptional control of ∆6-D and ∆5-D (Figure 1) [48,49]. The protection of FA from oxidation through RV as a chelator of copper and free-radical scavenger [64] or by interacting with the transcription factor Nrf2 [67,68] most likely adds to the effects of RV on ∆6-D. However, it should always be taken into consideration, that RV also interacts with other molecules and pathways (for example uptake of the FA into liver tissue [63], uptake of FA into cell organelles (e.g., acox1 [47]), the interaction with the hepatic sirtuin pathway [73,74], or modification of the biochemical pathways affecting adipogenesis in human adipocytes [72]) that can have indirect effects on the hepatic FA synthesis pathway. Thus, the observed effects of RV on rainbow trout in this study, are the result of many different interactions of RV on a molecular and physiological level, leading to the observed output of elevated EPA and DHA tissue levels.

Taken together, the presented data demonstrate an RV-mediated elevation of PUFAs that can putatively be ascribed to an elevation of hepatic ∆6-D protein levels and to the potential antioxidant properties of RV. However, the dietary administration of RV resulted in reduced feed intake in rainbow trout, which may be related to a lower palatability and warrants further research. Nevertheless, RV seems to be a promising dietary supplement that has the ability to increase LC-PUFA levels in farmed rainbow trout fed diets low in fish oil, potentially leading to enhanced fatty acid quality in aquaculture products. Further research should be conducted in other fish species to address the question of what extent RV may affect fatty acid profiles.

## 4. Materials and Methods

### 4.1. Experimental Diets and Housing Conditions of Rainbow Trout

Six different experimental diets (isonitrogenous and isoenergetic) were formulated on dry matter (DM) basis as shown in Table 5. All diets contained 10% of fish meal and consisted of equal amounts of mainly alternative plant protein sources (pea protein isolate, wheat gluten and rapeseed concentrate amongst others; Table 5). The experimental diets varied in the oil fraction, with decreasing fish oil contents (F4: 4%, F2: 2% and F0: 0% DM) and increasing vegetable oil contents (F4: 6.6%, F2: 8.6% and F0: 10.6% DM). Diet F4 served as an overall reference diet and accordingly as the control. Diet F2 contained half the amount of fish oil of diet F4 (50% substitution with plant oils) and was set at the requirement level of EPA + DHA in trout (0.4–0.5% DM of the diet [34]). Diet F0 completely lacked fish oil (100% substitution with plant oils) and served as the reference for a diet below the minimum requirement of EPA + DHA in rainbow trout. Fatty acid profiles and absolute amounts of EPA + DHA % DM of experimental diets are presented in Table 2. The three above-mentioned diets were referred to as basal diets (F4, F2, F0) and served as controls for similarly composited, but additionally with resveratrol (RV; *trans*-3,4′,5-trihydroxy stilbene, purity min. 98%, Chemos GmbH & Co. KG, Regenstauf, Germany) supplemented diets. Diets F4 + RV, F2 + RV and F0 + RV were supplemented with RV (0.3% DM, Table 5). The amino acid (AA) content of each diet was calculated based on the AA contents of single ingredients. All diets were formulated to meet the requirements for AA content in rainbow trout feed according to Rodehutscord et al. [83] and NRC [34]. 

A feeding trial with a total of 486 juvenile rainbow trout (Fischzucht Kortmann GbR, Hohenweststedt, Germany) was conducted at the facilities of the Gesellschaft für Marine Aquakultur (GMA) mbH in Büsum, Germany. All experiments were carried out according to the EU Directive 2010/63/EU for animal experiments and approved by the Ministry of Energy, Agriculture, the Environment, Nature and Digitalization (MELUND), Kiel, Germany (approved on 15 October 2014; project number: V244-7224.121.9-34). Trout were allowed to adapt to housing conditions prior to the experiment in a recirculating aquaculture system (7.6 m^3^, turnover rate 4 times h^−1^, moving bed biofilter and additional bead filter (PolyGeyser, Model DF-6, Aquaculture Systems Technologies, L.L.C., New Orleans, LA, USA), UV-light disinfection). Light regime was set at 14:10 h light:dark cycle during adaption and experimental treatment periods. Water quality parameters were determined daily and maintained in a suitable range for rainbow trout (7.93 pH, 15.1 ± 0.4 °C, 9.4 ± 0.3 mg/L O_2_, 0.2 ± 0.1 mg/L NH_4_, 6.9 ± 3.4 mg/L NO_2_ (Microquant test kit for NH_4_ and NO_2_, Merck, Darmstadt, Germany), 2.3 ± 0.7‰ PSU). Prior to experimental treatment, rainbow trout (mean body weight 36.35 ± 0.03 g) were randomized in six different groups in triplicate, each group with 27 individuals, and maintained in 18 tanks (150 L) of the experimental system. During the experimental period of eight weeks, fish were fed manually twice per day (8:30 a.m. and 4:30 p.m.) until apparent satiation.

### 4.2. Sampling

Samples were collected before the onset of the experimental period (day 0) and at the end of the experiment (day 58). For initial sampling at day 0, five individuals were sacrificed (pooled sample) and stored at −20 °C for determination of whole body fatty acid composition. In addition, five individuals were sacrificed for collection of liver samples. For mRNA quantification via qRT-PCR, one part of the liver tissue was preserved in RNALater (Sigma-Aldrich, Taufkirchen, Germany) and stored at −20 °C. For measurements of the protein levels via ELISA, a second part of the liver tissue was immediately shock-frozen and stored at −80 °C. For the determination of the liver lipid levels and the liver fatty acid composition, the remaining liver parts of five individuals were pooled into one sample and immediately stored at −80 °C. At the end of the feeding trial at day 58, corresponding samples for the determination of mRNA and protein levels were taken from five individuals per tank. Additionally, five individuals per tank were sacrificed (pooled samples) for analysis of whole body fatty acid composition. 

### 4.3. Lipid Extraction and Measurement of Fatty Acids

Extraction of total lipids and measurement of fatty acid methyl esters (FAMEs) was performed using a Gas Chromatograph with Flame Ionization Detector (GC-FID).

In brief, total lipids were extracted from liver samples according to Folch et al. [84]. Methylation of fatty acids and extraction of methylated FAMEs was conducted with the help of the Folch reagent (chloroform:methanol 2:1). Samples were neutralized using potassium hydroxide (0.1 M) and FAMEs were isolated by the addition of the Folch reagent and subsequent centrifugation for 10 min at 2000× *g*. The organic phase was collected and a second extraction with potassium hydroxide and the Folch reagent was performed, followed by centrifugation (5 min at 2000× *g*) and drying of samples under a N_2_ flux. Re-dissolved FAME samples were injected into a 7820A Agilent gas chromatograph (Agilent Technologies, Santa Clara, CA, USA) equipped with an Agilent HP-88 fused silica capillary column (60 m × 250 μm × 0.2 μm, Agilent Technologies) and helium (1.2 mL/min) as the carrier gas. The following temperature protocol was applied: initial temperature 125 °C, ramp 8 °C/min to 145 °C (26 min), ramp 2 °C/min to 220 °C (5 min). Chromatograms were recorded and analyzed using EZChrom Elite software (Agilent Technologies). FAME standards (11 FAMEs, see Table 2) were used to identify retention times of individual FAMEs. Fatty acid composition was calculated as a percentage of single FAME relative to total FAMEs. FAs as % DM of diet or mg/g tissue were calculated using 13:0 methyl ester as the internal standard. 

### 4.4. mRNA Extraction and qRT-PCR

Total mRNA was extracted from the trout liver samples using the Innuprep RNA Mini Kit (Analytik Jena, Jena, Germany) according to the manufacturer’s protocol. Tissue was homogenized in a TissueLyser II (Qiagen, Hilden, Germany) prior to RNA isolation. RNA concentration and purity were determined via NanoDrop measurements (NanoDrop2000c; ThermoScientific, Waltham, MA, USA) at 260, 280 and 230 nm absorbance. qRT-PCR was performed with a SensiFast SYBR No-ROX One-Step Kit (Bioline, London, UK) on a Rotor-Gene 6000 real-time PCR cycler (Corbett/Qiagen). Primers used and appropriate annealing temperatures are listed in Table 6. Transcript expression was absolutely quantified by calculating the input copy number using a standard curve. Subsequently, respective target mRNA expression levels of ∆6-D, PPARα, CPT1a and CPT1c were normalized to the mRNA expression level of the housekeeping gene elongation factor 1 α (EF1α). Data are shown as relative mRNA expression levels of respective target genes normalized to their internal control (EF1α) following absolute quantification (Figure 2).

### 4.5. Enzyme-Linked Immunosorbent Assay (ELISA)

Determination of ∆6-desaturase (∆6-D) protein levels was performed in liver tissue samples from rainbow trout using a Fish Fatty Acid Desaturase 2 ELISA Kit (MBS066226, MyBiosource Inc., San Diego, CA, USA; purchased from Biozol, Eching, Germany) according to the manufacturer’s instructions. In brief, tissue samples were weighted on dry ice and lysed in phosphate buffered saline using a TissueLyser II (Qiagen, Hilden, Germany). Following centrifugation, supernatants were applied to the Microelisa multiwall plate provided by the ∆6-D Kit. Samples were incubated with a HRP (horseradish peroxidase)-conjugate reagent followed by several washing steps. Protein concentration was quantified following HRP-mediated color reaction (consecutive application of Chromogen A, B and Stop solutions) by absorbance measurements at 450 nm using a Labsystems iEMS MF multiplate reader (MTX Lab Systems, Bradenton, FL, USA purchased from Thermo Fisher Scientific, Darmstadt, Germany). ∆6-D protein concentration was calculated using a standard curve. ∆6-D concentrations were normalized to total protein concentrations, which were evaluated via the Pierce bicinchoninic acid (BCA) kit (Thermo Fisher Scientific) according to the manufacturer’s protocol, respectively.

### 4.6. Statistical Analysis

All statistical analyses were performed using R (version 3.1.3) with an RStudio interface. The packages *gdata*, *multcomp*, *gplots*, *nparcomp*, *nlme*, and *piecewiseSEM* were used for graphical and statistical analysis. 

For FA composition, liver lipid levels, IBW, FBW, DFI, HSI and ∆6-D protein levels data evaluation started with the definition of an appropriate linear model. The data were proven to be normally distributed and homoscedastic based on a graphical residual analysis. The statistical model included the level of fish oil content (F4, F2, F0) and supplement (None, RV), as well as their interaction term. Based on this model, an analysis of variances (ANOVA) was conducted, followed by multiple contrast tests [85,86] to compare several levels of influencing factors, respectively.

For mRNA expression levels, the data evaluation was initiated with the definition of an appropriate mixed model [87,88] with fish oil content (F4, F2, F0), supplement type (None, RV) and their interaction term as fixed factors, and fish tank as random factor. A residual analysis revealed the data to be non-normally distributed. Multiple contrast tests for relative effects [86,89] were conducted to compare several levels of influencing factors, respectively.

## Figures and Tables

**Table 1:** Growth, feed intake (DFI) and hepatosomatic index (HSI) of rainbow trout fed the experimental diets. F4, F2 and F0 represent results which were obtained from fish fed the basal diets containing 4%, 2% and 0% DM fish oil. Basal diets were provided either in absence or presence of 0.3% DM resveratrol (+RV) for an 8-week feeding period.

	F4	F2	F0	F4 + RV	F2 + RV	F0 + RV
IBW ^1^	35.54 ± 0.20	35.34 ± 0.15	35.69 ± 0.42	35.66 ± 0.15	35.81 ± 0.18	35.63 ± 0.50
FBW ^2^	46.15 ± 1.26 ^m^	46.37 ± 0.18 ^x^	47.27 ± 2.19 ^a^	41.96 ± 0.90 ^n^	41.60 ± 1.15 ^y^	40.57 ± 1.62 ^b^
DFI ^3^	1.94 ± 0.07 ^m^	1.95 ± 0.06 ^x^	1.90 ± 0.11 ^a^	1.49 ± 0.04 ^n^	1.49 ± 0.07 ^y^	1.36 ± 0.08 ^b^
HSI ^4^	1.52 ± 0.40	1.49 ± 0.23	1.40 ± 0.16	1.32 ± 0.24	1.28 ± 0.30	1.51 ± 0.34

Values (mean ± SD, IBW, FBW and DFI: *n* = 3; HSI: *n* = 5) with different superscript letters within one row significantly differ with *p*-values < 0.05 based on ANOVA as described in Materials and methods. Superscript letters indicate the output of tests based on comparisons of supplement level within one fish oil inclusion group (a, b: F0 diets; x, y: F2 diets; m, n: F4 diets). ^1^ IBW = Initial body weight [g]; ^2^ FBW = Final body weight [g]; ^3^ DFI = Daily feed intake [% d^−1^]; ^4^ HSI = Hepatosomatic index [%] = (liver weight/final body weight) × 100.

**Table 2:** Fatty acid composition (in % of total fatty acid methyl esters (FAMEs) and in % DM of diet) of the experimental diets. F4, F2 and F0 are basal diets containing 4%, 2% and 0% DM fish oil. +RV indicates supplementation of basal diets with 0.3% DM resveratrol. The standard used for identification of individual FAMEs consisted of all 11 FAMEs shown here.

[% of FAMEs]	F4	F2	F0	F4 + RV	F2 + RV	F0 + RV
14:0	1.79	1.04	0.70	1.77	1.04	0.35
16:0	11.24	9.75	9.76	11.22	9.74	8.89
18:0	3.58	3.18	3.17	3.55	3.11	2.95
**Σ SFA ^a^**	**16.60**	**13.97**	**13.64**	**16.54**	**13.90**	**12.20**
16:1	1.72	1.09	0.83	1.73	1.08	0.52
18:1n-9c	33.46	40.03	42.00	33.38	39.67	45.18
18:1n-7c	2.65	2.92	3.14	2.68	2.99	3.21
**Σ** **MUFA ^b^**	**37.83**	**44.04**	**45.95**	**37.79**	**43.74**	**48.91**
18:2n-6c	28.83	28.26	27.90	28.87	28.61	27.79
18:3n-3	10.95	10.11	9.29	11.00	10.07	8.93
20:5n-3	2.28	1.44	1.20	2.35	1.40	0.68
22:5n-3	0.49	0.18	0.27	0.32	0.33	0.21
22:6n-3	3.02	2.00	1.80	3.12	1.96	1.27
**Σ** **PUFA ^c^**	**45.57**	**41.99**	**40.42**	**45.67**	**42.37**	**38.89**
**Σ** **EPA + DHA ^d^**	**5.30**	**3.43**	**2.35**	**5.47**	**3.36**	**1.96**
EPA/DHA	0.75	0.72	0.61	0.75	0.72	0.54
18:3n-3/18:2n-6	0.38	0.36	0.33	0.38	0.35	0.32
**Σ** **EPA + DHA % DM ^e^**	**0.74**	**0.56**	**0.34**	**0.91**	**0.56**	**0.26**

^a^ ∑ SFA is the sum of saturated fatty acids; ^b^ ∑ MUFA is the sum of monounsaturated fatty acids; ^c^ ∑ PUFA is the sum of n-3 and n-6 polyunsaturated fatty acids; ^d^ ∑ EPA + DHA is the sum of eicosapentaenoic acid (20:5n-3) and docosahexaenoic acid (22:6n-3); ^e^ Determination of EPA + DHA % DM of diet was done using the internal standard 13:0 methyl ester and amount of lipid measured in the diet (Table 5).

**Table 3:** Fatty acid composition (in % of total fatty acid methyl esters (FAMEs)) of whole body homogenates of rainbow trout before (Initial) and at the end of the feeding trial. F4, F2, and F0 represent results which were obtained from fish fed the basal diets containing 4%, 2% and 0% DM fish oil. Supplementary designation with +RV indicates that fish were fed the diets supplemented with 0.3% DM resveratrol (+RV). The standard used for identification of individual FAMEs consisted of all 11 FAMEs shown here.

[% of FAMEs]	Initial	F4	F2	F0	F4 + RV	F2 + RV	F0 + RV
14:0	4.97	3.67 ± 0.29	3.29 ± 0.49	3.47 ± 0.26 ^a^	3.19 ± 0.32 ^A^	2.47 ± 0.19 ^A,B^	1.73 ± 0.69 ^b,B^
16:0	17.73	19.44 ± 0.84	20.15 ± 1.50	19.26 ± 0.52	18.54 ± 1.62	19.57 ± 0.47	20.16 ± 0.63
18:0	4.31	5.78 ± 0.48	6.26 ± 0.73	5.95 ± 0.47 ^b^	5.73 ± 0.96 ^B^	6.59 ± 0.34 ^A,B^	7.96 ± 0.73 ^a,A^
**Σ** **SFA ^a^**	**27.01**	**28.89 ± 1.05**	**29.70 ± 2.09**	**28.68 ± 0.73**	**27.46 ± 2.71**	**28.63 ± 0.65**	**29.85 ± 0.71**
16:1	5.05	4.18 ± 0.52	3.70 ± 0.32 ^x^	3.75 ± 0.29 ^a^	3.50 ± 0.41 ^A^	2.71 ± 0.13 ^y,A,B^	2.15 ± 0.69 ^b,B^
18:1n-9c	27.25	25.03 ± 0.91	23.94 ± 3.65	25.88 ± 1.77 ^a^	27.46 ± 6.18	20.48 ± 0.53	17.91 ± 1.30 ^b^
18:1n-7c	3.36	3.12 ± 0.02	3.10 ± 0.03 ^+^	3.17 ± 0.11 ^a^	3.17 ± 0.21 ^A^	2.8 ± 0.08 ^A,+^	2.48 ± 0.25 ^b,B^
**Σ** **MUFA ^b^**	**35.66**	**32.33 ± 1.34**	**30.74 ± 3.72**	**32.80 ± 2.11 ^a^**	**34.13 ± 6.61 ^A^**	**26.00 ± 0.61 ^B^**	**22.55 ± 2.24 ^b,B^**
18:2n-6c	14.11	11.81 ± 1.63	10.31 ± 2.67	11.41 ± 1.13 ^a^	12.50 ± 3.44 ^A^	8.72 ± 0.12 ^A,B^	5.46 ± 1.63 ^b,B^
18:3n-3	3.44	2.28 ± 0.35	1.92 ± 0.47	2.02 ± 0.21 ^a^	2.66 ± 0.73 ^A^	1.46 ± 0.10 ^B^	0.97 ± 0.26 ^b,B^
20:5n-3	4.29	3.91 ± 0.10	4.03 ± 0.63	3.74 ± 0.28 ^b^	3.65 ± 1.13 ^B^	4.68 ± 0.20 ^A,B^	5.54 ± 0.41 ^a,A^
22:5n-3	1.97	2.24 ± 0.05	2.28 ± 0.40	2.11 ± 0.12 ^+^	1.95 ± 0.71 ^B^	2.57 ± 0.04 ^A,B^	2.83 ± 0.21 ^A,+^
22:6n-3	13.53	18.55 ± 2.09	21.02 ± 3.88 ^+^	19.24 ± 2.33 ^b^	17.65 ± 6.16 ^B^	27.95 ± 0.84 ^A,+^	32.81 ± 3.11 ^a,A^
**Σ** **PUFA ^c^**	**37.34**	**38.78 ± 0.65**	**39.56 ± 1.74 ^y^**	**38.52 ± 1.39 ^b^**	**38.41 ± 3.91 ^B^**	**45.38 ± 0.93 ^x,A^**	**47.60 ± 1.55 ^a,A^**
Σ EPA + DHA ^d^	17.82	22.46 ± 2.19	25.05 ± 4.48	22.98 ± 2.60 ^b^	21.29 ± 7.29 ^B^	32.63 ± 0.89 ^A^	38.35 ± 3.38 ^a,A^
EPA/DHA	0.32	0.21 ± 0.02	0.19 ± 0.01 ^+^	0.20 ± 0.01 ^++^	0.21 ± 0.01 ^A^	0.17 ± 0.01 ^B,+^	0.17 ± 0.01 ^B,++^
Σ n-3/Ʃ n-6	1.65	2.33 ± 0.45	3.03 ± 1.14	2.42 ± 0.57 ^b^	2.34 ± 1.45 ^B^	4.20 ± 0.07 ^B^	8.24 ± 2.61 ^a,A^

^a^ ∑ SFA is the sum of saturated fatty acids; ^b^ ∑ MUFA is the sum of monounsaturated fatty acids; ^c^ ∑ PUFA is the sum of n-3 and n-6 polyunsaturated fatty acids; ^d^ ∑ EPA + DHA is the sum of eicosapentaenoic acid (20:5n-3) and docosahexaenoic acid (22:6n-3). Values (mean ± SD, *n* = 3, Initial: *n* = 1) with different superscript letters within one row significantly differ with *p*-values < 0.05 based on ANOVA as described in Materials and methods. Tests are based on comparison of supplement type within one fish oil inclusion level (a, b: F0 diets; x, y: F2 diets) or comparisons of fish oil inclusion level within +RV supplemented groups (A, B). ^+^/^++^ within one row indicates a statistical tendency between similarly marked groups with a *p*-value < 0.1.

**Table 4:** Total fat (mg/g liver) and fatty acid composition (in % of total fatty acid methyl esters (FAMEs) and in mg/g liver) of liver tissue of rainbow trout before (Initial) and at the end of the feeding trial. F4, F2, and F0 represent results which were obtained from fish fed the basal diets containing 4%, 2% and 0% DM fish oil. Supplementary designation with +RV indicates that fish were fed the diets supplemented with 0.3% DM resveratrol (+RV). The standard used for identification of individual FAMEs consisted of all 11 FAMEs shown here.

	Initial	F4	F2	F0	F4 + RV	F2 + RV	F0 + RV
**Total fat [mg/g]**	13.60	16.70 ± 1.56 ^N,n^	21.47 ± 3.10 ^x^	26.80 ± 2.55 ^M^	24.80 ± 1.06 ^m^	30.08 ± 3.11 ^y^	26.70 ± 0.99
**FA [% of FAMEs]**							
14:0	1.63	1.45 ± 0.19	1.24 ± 0.13	1.33 ± 0.15	1.32 ± 0.08	1.4 ± 0.15	1.33 ± 0.11
16:0	20.75	22.80 ± 1.01	24.07 ± 1.35	24.30 ± 1.01 ^a^	23.01 ± 0.6	22.89 ± 2.61	21.28 ± 0.71 ^b^
18:0	6.90	8.14 ± 0.59	8.66 ± 0.58	8.15 ± 0.38	7.74 ± 0.38	7.52 ± 1.48	7.12 ± 0.18
**Σ SFA ^a^**	**29.28**	**32.39 ± 1.22**	**33.96 ± 1.79**	**33.78 ± 1.11 ^a^**	**32.06 ± 0.89**	**31.81 ± 3.95**	**29.74 ± 0.79 ^b^**
16:1	2.20	1.64 ± 0.21	1.46 ± 0.25	1.92 ± 0.33	1.56 ± 0.25	1.91 ± 0.53	1.70 ± 0.32
18:1n9c	14.93	18.60 ± 4.28	15.14 ± 1.39	14.68 ± 1.00	14.58 ± 0.78	15.61 ± 1.24	16.40 ± 1.64
18:1n7c	2.37	2.55 ± 0.29	2.36 ± 0.23	2.66 ± 0.46	2.46 ± 0.33	2.72 ± 0.37	2.73 ± 0.29
**Σ MUFA ^b^**	**19.50**	**22.79 ± 4.73**	**18.96 ± 1.86**	**19.25 ± 1.62**	**18.60 ± 1.23**	**20.25 ± 2.13**	**20.83 ± 2.19**
18:2n6c	8.17	6.09 ± 0.99	6.05 ± 0.99	5.74 ± 0.27	6.27 ± 0.62	5.73 ± 0.52	6.33 ± 0.35
18:3n3	1.45	0.69 ± 0.24	0.56 ± 0.16	0.47 ± 0.11	0.58 ± 0.08	0.57 ± 0.13	0.57 ± 0.20
20:5n3	4.59	3.90 ± 0.25	3.55 ± 0.37	3.65 ± 0.76	4.43 ± 0.41	4.37 ± 0.81	4.61 ± 0.64
22:5n3	1.87	1.87 ± 0.21 ^+^	1.88 ± 0.18	1.89 ± 0.22 ^b^	2.30 ± 0.23 ^+^	2.22 ± 0.22	2.47 ± 0.19 ^a^
22:6n3	35.13	32.26 ± 4.40	35.03 ± 1.48	35.21 ± 1.18	35.77 ± 0.93	35.06 ± 1.71	35.45 ± 1.89
**Σ PUFA ^c^**	**51.21**	**44.81 ± 3.51**	**47.08 ± 0.21**	**46.97 ± 1.96**	**49.34 ± 0.72**	**47.95 ± 2.49**	**49.43 ± 1.79**
Σ EPA + DHA ^d^	39.72	36.16 ± 4.50	38.58 ± 1.43	38.86 ± 1.68	40.20 ± 0.63	39.44 ± 2.46	40.06 ± 1.77
EPA/DHA	0.13	0.12 ± 0.02	0.10 ± 0.01	0.10 ± 0.02	0.12 ± 0.01	0.12 ± 0.02	0.13 ± 0.02
Σ n-3/Σ n-6	5.27	6.54 ± 1.67	6.92 ± 1.31	7.19 ± 0.45	6.92 ± 0.77	7.37 ± 1.03	6.83 ± 0.60
EPA mg/g	0.58	0.72 ± 0.24 ^+^	0.70 ± 0.03 ^y^	0.83 ± 0.28 ^b,+^	1.02 ± 0.08	1.04 ± 0.39 ^x^	1.29 ± 0.23 ^a^
DHA mg/g	4.44	4.79 ± 1.19 ^N^	7.00 ± 1.18 ^y^	7.86 ± 1.52 ^b,M^	8.25 ± 0.42	10.20 ± 0.68 ^x^	11.08 ± 2.75 ^a^
Σ EPA + DHA mg/g ^e^	5.02	5.51 ± 1.23 ^N^	7.71 ± 1.20 ^y^	8.69 ± 1.78 ^b,M^	9.27 ± 0.37	11.24 ± 0.78 ^x^	12.38 ± 2.93 ^a^

^a^ ∑ SFA is the sum of saturated fatty acids; ^b^ ∑ MUFA is the sum of monounsaturated fatty acids; ^c^ ∑ PUFA is the sum of n-3 and n-6 polyunsaturated fatty acids; ^d^ ∑ EPA + DHA is the sum of eicosapentaenoic acid (20:5n-3) and docosahexaenoic acid (22:6n-3); ^e^ Determination of EPA + DHA mg/g liver tissue was done using the internal standard 13:0 methyl ester and amount of total fat in the liver. Values (mean ± SD, *n* = 3, Initial: *n* = 1) with different superscript letters within one row significantly differ with *p*-values < 0.05 based on ANOVA as described in Materials and methods. Tests are based on comparison of supplement type within one fish oil inclusion level (a, b: F0 diets; x, y: F2 diets; m, n F4 diets) or comparisons of fish oil inclusion level (M, N). ^+^ within one row indicates a statistical tendency between similarly marked groups with a *p*-value < 0.1.

**Table 5:** Ingredients and nutrient composition (in % of dry matter (DM)) of the experimental diets. F4, F2 and F0 are basal diets containing 4%, 2% and 0% DM fish oil. +RV indicates supplementation of basal diets with 0.3% DM resveratrol.

Ingredients [% DM]	F4	F2	F0	F4 + RV	F2 + RV	F0 + RV
Fish meal (*Clupea* sp.) ^a^	10	10	10	10	10	10
Casein ^b^	5.9	5.9	5.9	5.9	5.9	5.9
Rapeseed concentrate ^c^	13	13	13	13	13	13
Pea protein isolate ^d^	13.58	13.58	13.58	13.58	13.58	13.58
Wheat gluten ^e^	17.95	17.95	17.95	17.95	17.95	17.95
Wheat starch ^e^	20	20	20	20	20	20
**Fish oil ^a^**	**4**	**2**		**4**	**2**	
Linseed oil ^f^	1.50	0.94	0.38	1.50	0.94	0.38
Rapeseed oil ^g^	3.19	6.11	9.04	3.19	6.11	9.04
Sunflower oil ^g^	1.91	1.55	1.18	1.91	1.55	1.18
Vitamin mineral premix ^h^	0.5	0.5	0.5	0.5	0.5	0.5
Lysine ^i^	0.7	0.7	0.7	0.7	0.7	0.7
Dicalcium-phosphate ^j^	1	1	1	1	1	1
Inert filler ^k^	6.77	6.77	6.77	6.77	6.77	6.77
**Resveratrol (RV) ^l^**				**0.3**	**0.3**	**0.3**
Nutrient composition [% DM]						
Dry matter (in % of diet)	93.2	93.1	93.2	93.3	92.8	91.7
Crude protein	50.1	50.3	50.2	50.0	50.3	50.8
Crude lipid	14.9	15.1	15.1	15.0	15.0	15.0
Crude ash	6.4	6.4	6.4	6.4	6.4	6.2
Total KOH ^m^	28.6	28.2	28.4	28.6	28.3	28.0
Gross energy [MJ kg^−1^ DM]	22.72	22.72	22.70	22.75	22.75	22.93

^a^ Vereinigte Fischmehlwerke Cuxhaven GmbH & Co. KG, Cuxhaven, Germany; ^b^ Molkerei MEGGLE Wasserburg GmbH & Co. KG, Wasserburg, Germany; ^c^ BioExx Speciality Proteins LTD, Toronto, ON, Canada; ^d^ Emsland-Stärke GmbH, Emlichheim, Germany; ^e^ KRÖNER STÄRKE GmbH, Ibbenbüren, Germany; ^f^ Makana Produktion und Vertrieb GmbH, Offenbach a.d. Queich, Germany; ^g^ Different food stores, Büsum, Germany; ^h^ Emsland-Aller Aqua GmbH, Golßen, Germany; ^i^ Biolys, Evonik Industries AG, Essen, Germany; ^j^ Lehmann & Voss & Co (LuV), Hamburg, Germany; ^k^ Carboxy-methyl-cellulose (CMC), Mikro-Technik GmbH & Co. KG, Bürgstadt/Main, Germany; ^l^ CHEMOS GmbH & Co. KG, Regenstauf, Germany; ^m^ Total KOH: total carbohydrates = 1000 − (crude protein + crude fat + crude ash).

**Table 6:** Primer sequences for hepatic mRNA measurements via qRT-PCR. Forward and reverse primers as well as their specific annealing temperatures used for qRT-PCR measurements of mRNA levels in total RNA samples extracted from rainbow trout liver.

Primer	Sequence 5′ → 3′	Annealing Temperature (°C)
**∆6-D** ^a,^* forward	GCTGGAGARGATGCCACGGA	61
**∆6-D** ^a,^* reverse	TGCCAGCTCTCCAATCAGCA	61
**EF1α** ^b,^* forward	ACAAGCCCCTYCGTCTGCC	61
**EF1α** ^b,^* reverse	GCATCTCCACAGACTTSACCTCAG	61
**PPARα** ^c,§^ forward	CTGGAGCTGGATGACAGTGA	55
**PPARα** ^c,§^ reverse	GGCAAGTTTTTGCAGCAGAT	55
**CPT1a** ^d,§^ forward	TCGATTTTCAAGGGTCTTCG	55
**CPT1a** ^d,§^ reverse	CACAACGATCAGCAAACTGG	55
**CPT1c** ^d,§^ forward	CGCTTCAAGAATGGGGTGAT	59
**CPT1c** ^d,§^ reverse	CAACCACCTGCTGTTTCTCA	59

^a^ ∆6-D: ∆6-desaturase; ^b^ EF1α: Elongation factor 1 α; ^c^ PPARα: Peroxisome proliferator-activated receptor α; ^d^ CPT1: Carnitine palmitoyl transferase 1; * Geay et al. (2012); ^§^ Kolditz et al. (2008).
